# The potentiating effect of intravenous dexamethasone upon preemptive pudendal block analgesia for hypospadias surgery in children managed with Snodgrass technique: a randomized controlled study: Dexamethasone for pain management in children

**DOI:** 10.1186/s12871-024-02536-3

**Published:** 2024-04-16

**Authors:** Sonia Ben Khalifa, Ahmed Ben Slimene, Hajer Blaiti, Refka Kaddour, Amjed Fekih Hassen, Pierre Pardessus, Christopher Brasher, Souhayl Dahmani

**Affiliations:** 1 Department of Anesthesia and Intensive Care, Robert Ballanger Hospital, 1 boulevard Robert Ballanger, Aulnay-Sous-Bois, 93602 France; 2 Department of Anesthesia and Intensive Care, Children Hospital, Boulevard 9 avril, Baab Saadoun, Tunis, Tunisia; 3 Université de Paris-Cité, Paris, France; 4 Department of Anesthesia and Intensive Care, Robert Debré Hospital, 48 boulevard Sérurier, Paris, 75019 France; 5 FHU I2D2. Robert Debré Hospital, 48 boulevard Sérurier, Paris, 75019 France; 6 Department of Anesthesia & Pain Management, Royal Children’s Hospital, Melbourne, Australia; 7 Anesthesia and Pain Management Research Group, Murdoch Children’s Research Institute, Parkville, Australia; 8 Department for Integrated Critical Care, University of Melbourne, Melbourne, Australia

**Keywords:** Dexamethasone, Pudendal block, Children, Hypospadias

## Abstract

**Introduction:**

Evidence regarding the potentiating effects of intravenous dexamethasone on peripheral regional anesthesia in children is sparse. The objective of the current study was to investigate the potentiating effect of intravenous dexamethasone upon pudendal block during surgical correction of hypospadias using Snodgrass technique.

**Methods:**

The study consisted of a monocentric, randomized controlled, double-blinded study. Patients were randomized to receive either intravenous dexamethasone 0.15 mg.kg^− 1^ (D group) or a control solution (C group). Both groups received standardized anesthesia including a preemptive pudendal block performed after the induction of anesthesia. The primary outcome was the proportion of patients needing rescue analgesia. Secondary outcomes were other pain outcomes over the first 24 postoperative hours.

**Results:**

Overall, 70 patients were included in the study. Age were 24 [24; 36] and 26 [24; 38] months in the D and C groups, respectively (*p* = 0.4). Durations of surgery were similar in both groups (60 [30; 60], *p* = 1). The proportion of patients requiring rescue analgesia was decreased in the D group (23% versus 49%, in D and C groups respectively, *p* = 0.02). The first administration of rescue analgesia was significantly delayed in the D group. Postoperative pain was improved in the D group between 6 and 24 h after surgery. Opioid requirements and the incidence of vomiting did not significantly differ between groups.

**Conclusion:**

Associating intravenous dexamethasone (0.15 mg.kg^− 1^) to pudendal block during hypospadias surgery improves pain control over the first postoperative day. Further studies are needed in order to confirm these results.

**ClinicalTrials.gov Identifier:**

NCT03902249.

**A. What is already known:**

dexamethasone has been found to potentiate analgesia obtained with regional anesthesia in children.

**B. What this article adds:**

intravenous dexamethasone was found to improve analgesia with a preemptive pudendal block during hypospadias surgery.

**C. Implications for translation:**

results of this study indicate that intravenous dexamethasone could be used as an adjunct to pudendal block.

**Supplementary Information:**

The online version contains supplementary material available at 10.1186/s12871-024-02536-3.

## Introduction

Over the last 2 decades, great progress has been made in the both systemic and regional postoperative pain management [1]. However, despite these major advances, postoperative pain is still undertreated and this results in patient dissatisfaction [2].

Such paradoxical findings are probably related to many factors, although the underuse of available techniques for pain management [2] and the reduction in available human resources [3] are likely to be key factors. As such, any simple technique allowing for prolonged analgesia is welcome. This is especially the case for urological surgeries such as hypospadias because of anxiety for both patients and caregivers when providing postoperative pain.

Regional anesthesia and analgesia for pediatric surgery continues to progress. Numerous studies have demonstrated improved intraoperative and postoperative analgesia using regional techniques [1, 4, 5]. In addition, many adjuncts to regional anesthesia, namely clonidine, dexmedetomidine and dexamethasone, have been shown to improve pain relieve quality and prolong the duration of postoperative analgesia without rescue [6]. Among these techniques, pudendal block has been shown as a valuable regional anesthesia technique for pain management after hypospadias surgery [7, 8]. Specifically, it has been demonstrated as allowing the same quality of analgesia as caudal block with a reduced need for rescue analgesia.

Dexamethasone has been shown to potentiate the quality and the duration of regional analgesia either when administered by regional or intravenous route in adult patients [9]. However, results in pediatric studies have varied depending on the employed regional anesthesia technique [10–14]. Moreover, optimal dexamethasone dosage to potentiate local anesthesia effect is still the subject of debate [11].

The goal of this study was to investigate the efficacy of preemptive intravenous dexamethasone (0.15 mg.kg^− 1^) in the potentiation of pudendal block for pain management during hypospadias surgery in children. The primary outcome was the proportion of patients needing rescue analgesia during the first 24 postoperative hours. Secondary outcomes were: (a) the quality of analgesia during the first 24 postoperative hours (a decrease of pain intensity), (b) postoperative opioid requirements over the first 24 postoperative hours and (c) the number of episodes of vomiting during the first 24 postoperative hours.

## Materials and methods

This study consists of a randomized, double blinded study. It was performed in the Bechir-Hamza Children’s Hospital, Tunis, Tunisia. It was approved by our institutional review board (Comité de protection des personnes, Hôpital, d’enfants Béchir-Hamza; # 123–2017, Chairman: Professor Said JLIDI). Informed written consent was obtained from all parents. Reporting of the current study follows the consort checklist, and the trial was registered (ClinicalTrials.gov Identifier: NCT03902249, registration on 04/04/2019).

### Inclusion and exclusion criteria

The inclusion criteria selected patients aged < 18 years of age, American Society of Anesthesiologists health status (ASA) 1 to 2, undergoing surgical correction of hypospadias (all forms) and Snodgrass technique for surgery management. Exclusion criteria included: refusal of study participation and/or personal data use, coagulation disorder or skin infection at the puncture point (for regional anesthesia), preoperative administration of corticosteroids for other reasons, known allergic reactions to any medication used during the study (anesthetics agents, opioids and common non-opioid analgesics).

Patients were excluded from the study (and from the analyses) in case of failure of regional anesthesia or any intraoperative complication related to regional anesthesia (systemic toxicity, hematoma or regional infection).

### Randomization and masking

The patients were assigned to the control or intervention groups using the opaque sealed envelope technique based on computer-generated randomized numbers in blocks of 5 patients per group.

Dexamethasone was prepared by hospital pharmacists. Four mg of dexamethasone was diluted in 8 ml of normal saline solution (final concentration 0.5 mg.ml^− 1^). The volume to be injected was 0.3.(weight in kg) ml. The control group received 0.9% saline solution presented in the same packaging. Patients and all healthcare professional involved in patients’ management were unaware of the assigned treatment.

### Perioperative anesthesia management

Anesthesia was standardized in all patients. Pre-operatively, no premedication was administered; all patients were allowed to drink apple juice and water freely until 2 h before surgery. After preoxygenation, anesthesia induction was performed with sevoflurane 6% in an O_2_/air 50%/50% mixture. After securing intravenous access, fentanyl 3 mcg.kg^− 1^ and either dexamethasone or the control solution was administered. The airway was secured using a supraglottic device (igel, Intersurgical®, Wokingham, Berkshire, UK) and controlled ventilation was performed. A tidal volume of 7 to 9 ml.kg^− 1^ of an O_2_/Air mixture (50%/50%) was administered, an end-expiratory pressure 5 cmH_2_O was applied, and respiratory rate was adjusted to an end-tidal CO_2_ of 35 to 40 mmHg. The pudendal block was performed after the induction of anesthesia (and dexamethasone or the control solution adminitsration) and before incision. Hypnosis was achieved employing 1 age-adjusted minimal alveolar concentration of sevoflurane. Any increase in blood pressure or heart rate > 20% in comparison to preoperative values was treated by a fentanyl bolus of 3 mcg.kg^− 1^. Intraoperative maintenance fluid management consisted of Ringer’s Lactate administered according to the Holliday and Segar formula [15]. Body temperature was maintained between 36.5° and 37 °C using a simple warmer system on the upper part of the body.

The pudendal block was performed with a nerve stimulator under aseptic conditions [7, 8]. Patients were positioned in the lithotomy position. The point of injection was the medial side of the inferomedial ischial tuberosity, located at the 3- and 9-o’clock positions relative to the anus. With initial settings of 1.5 mA and 2 Hz, the nerve stimulator needle (22 gauge Stimuplex A, 50–100 mm; B. Braun, Melsungen, Germany) was inserted perpendicular to the skin, observing for the external anal sphincter contraction. The needle was advanced cranially, until obtaining penile movement. Maintenance of penile movement with reduction of stimulator current to 0.3 to 0.4 mA confirmed adequate proximity to the pudendal nerve. After negative blood aspiration, 0.1 ml.kg^− 1^ of 0.5% bupivacaine solution without epinephrine was injected per side. Adequate analgesia provided by the pudendal block was judged by the stability of the increase in heart rate or blood pressure < 20% in comparison to preoperative values during surgery.

Postoperatively, intravenous fluid administration consisted of a balanced crystalloid solution at 2 ml.kg^− 1^.h^− 1^ until the first postoperative day (24 h). Postoperative analgesia was standardized. Pain was evaluated using the CHEOPS score at arrival in the PACU (H0) at 30 min, and at 1, 2, 3, 4, 8 and 24 h after admission to PACU. In case of significant pain (CHEOPS > 7), a paracetamol bolus was administered (15 mg.kg^− 1^). In case of persistent pain 30 min after paracetamol administration, an intravenous bolus of nalbuphine (0.2 mg.kg^− 1^) was administered. All patients were managed in the hospital during 24 h and discharged the day after surgery.

### Surgical technique

All procedures were performed using the Snodgrass technique.

### Data collected

Data collected and analyzed consist of: age, weight, ASA status, type of hypospadias, the duration of the surgery, the time for the first rescue analgesia (paracetamol and nalbuphine), the number of boluses of each medication, the CHEOPS scores during the first 24 postoperative hours after surgery (admission to the PACU then 30 min, 1 h, 2 h, 3 h, 4 h, 8 h, 12 h and 24 h after admission to the PACU), the occurrence of vomiting during the 24 postoperative hours. The primary outcome was the proportion of patients needing any rescue analgesia during the first 24 postoperative hours. Secondary outcomes were: (a) the delay for the first administration of rescue analgesia, (b) postoperative CHOPS scores (c) opioid requirements during the first 24 postoperative hours and (d) the number of episodes of vomiting during the first 24 postoperative hours.

Long term follow-up after surgery explored surgical complications (urethrocutaneous fistula, meatal stenosis, urethral stricture, urethral diverticulum, glans dehiscence, breakdown, and cosmetic unfavorable outcome requiring redo-surgery) [16], Cometic result and micturition functions.

### Statistical analysis

Data were expressed as median and [25–75 interquartile ranges] for continuous variables. Comparisons were performed using the Mann and Whitney test, the X², Fisher’s exact test and the Kaplan-Meyer survival curve.

The sample size for the current study was calculated according to previous studies on the efficacy of pudendal block during hypospadias surgery. Studies found rescue analgesia rates in pudendal block groups to range from 20% [8] to 7% [7]. Given that previous studies demonstrate a reduction in rescue analgesia with dexamethasone use [9, 11, 14], we hypothesized that rescue analgesia would be required in 20% of control group patients and 0% of dexamethasone group patients. Accordingly, 34 patients per group were necessary to ensure an alpha risk of 5% and a power of 80% 1. Taking into account for a small number of excluded patients for block failure, we designed for 36 patients per arm.

Statistical analyses were performed using SPSS 20.0 software (IBM Company, Chicago, Illinois, USA). P value was set to 0.05 and Bonferroni correction was applied to multiple comparisons. Results were expressed as N (%) or median [25; 75 interquartile ranges].

## Results

**Fig. 1:**
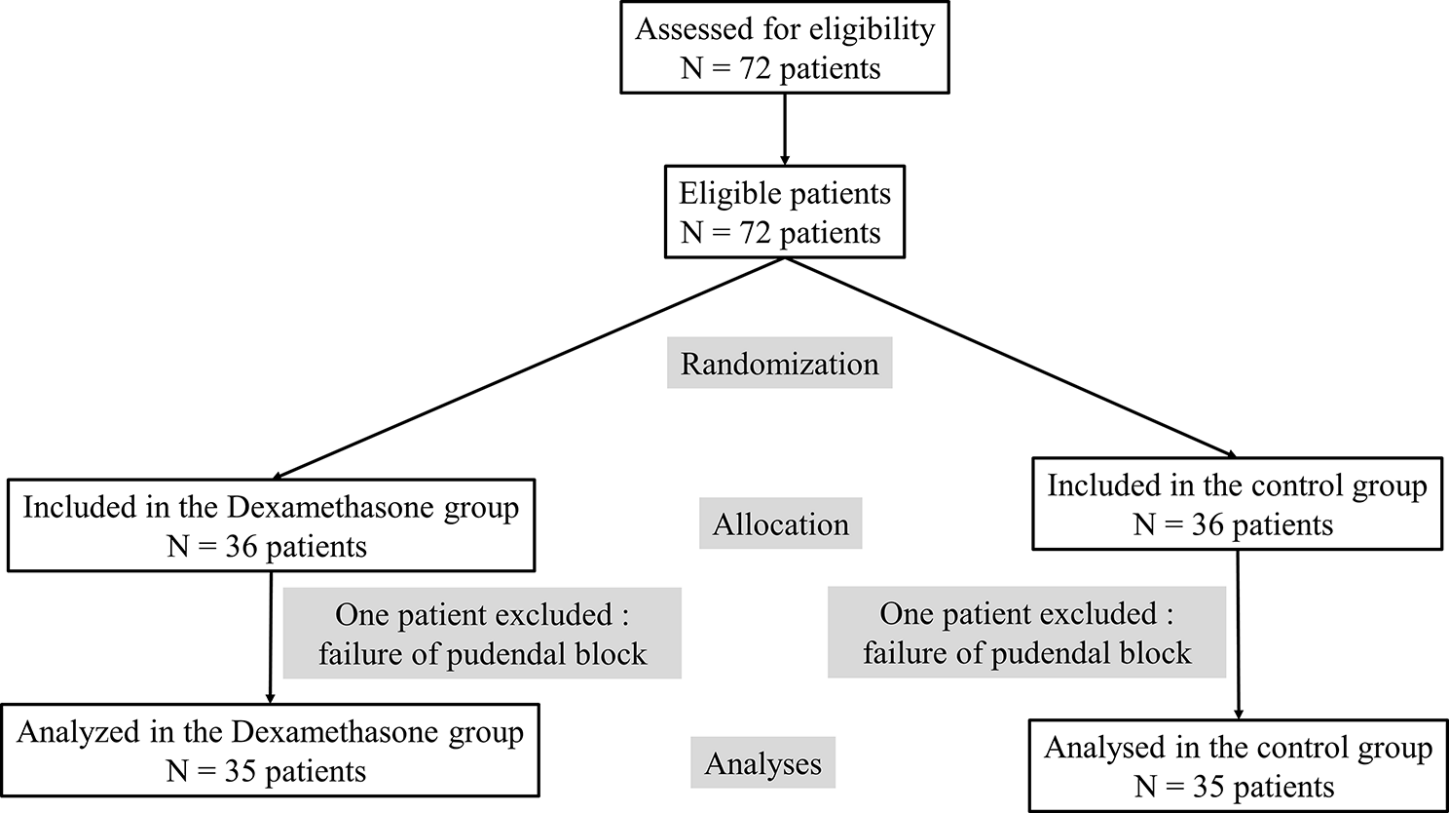
Flowchart of included patients

Over a period of two years (from January 2018 to December 2019), 72 patients were eligible for the study, and all were enrolled. Two patients were excluded because of pudendal block failure (Fig. 1). All included patient did not receive an additional bolus of fentanyl. No other block complications were observed.

**Fig. 2:**
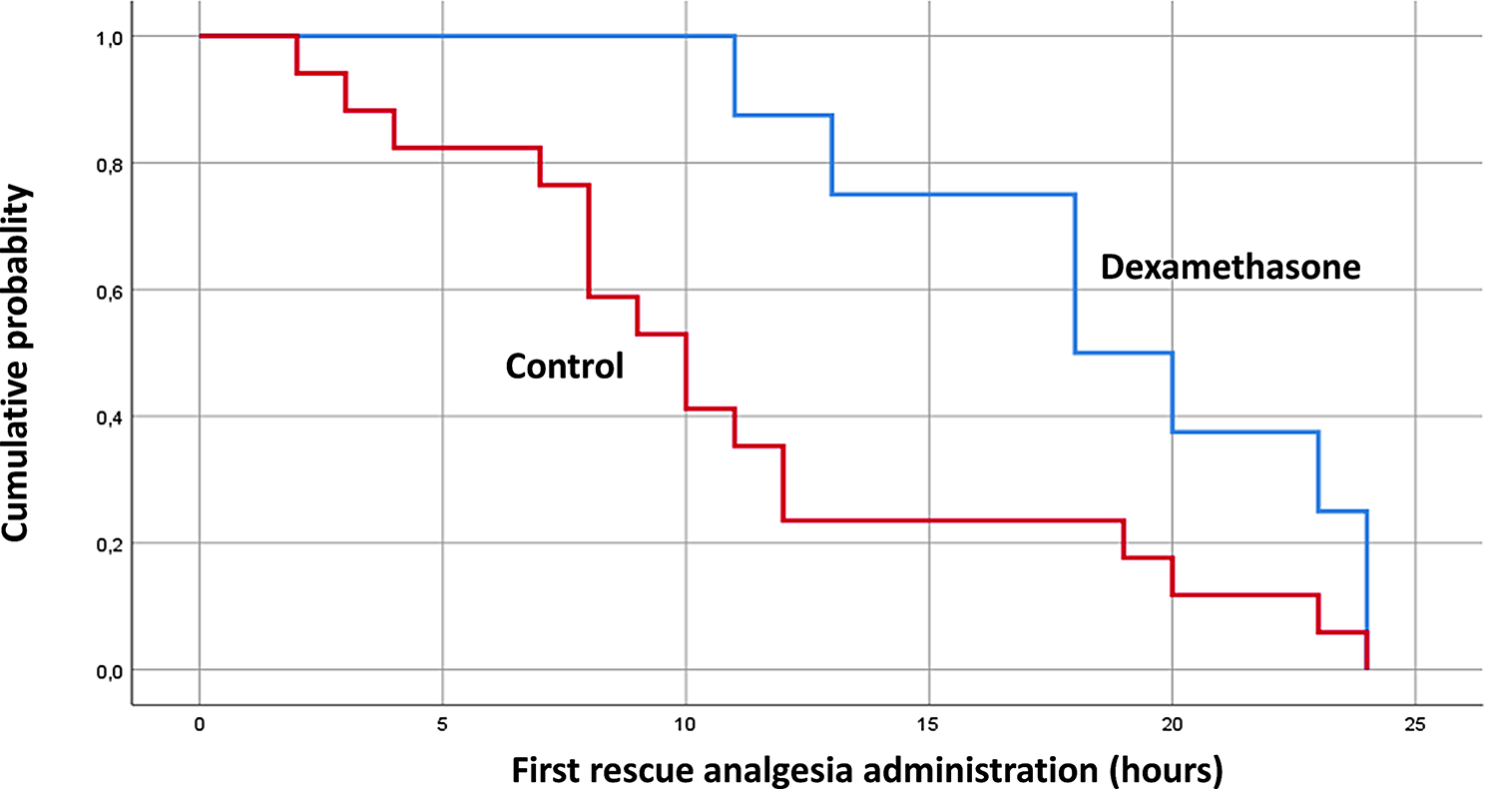
Kaplan-Mayer curve of the time of the first administration of rescue analgesia

**Fig. 3:**
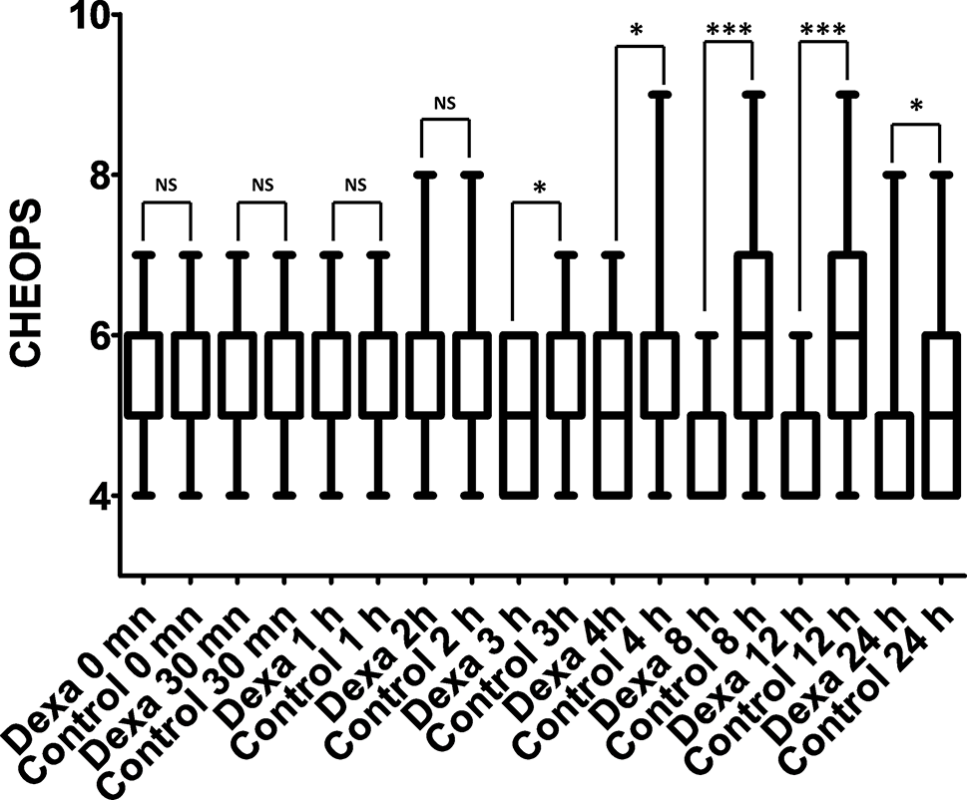
Pain scores in the Dexamethasone (Dexa) and control group during the first postoperative day

Demographic characteristics, health status and surgical details are displayed in Table 1. The proportion of patients requiring any rescue analgesia was significantly decreased in the dexamethasone group (Table 2: 8 (23%) versus 17 (49%) for the control group; *p* = 0.02). Furthermore, the time to any first administration of rescue analgesia was significantly delayed in the dexamethasone group (Table 2; Fig. 2). Pain intensity (CHEOPS score) was decreased in the dexamethasone group from the third to the 24th hour after surgery (Fig. 3). Although there was a tendency toward a reduced opioid requirement, the comparison did not reach statistical significance. There was no significant difference in postoperative vomiting between the two study groups.

**Table 1:** Description of the two samples of patients. Data are expressed as median [25–75 interquartile ranges] or N (%)

	Dexamethasone group (*N* = 35)	Control group (*N* = 35)	P value
Age (months)	24 [24; 36]	26 [24; 38]	0.4
Weigh (kg)	14 [12; 14]	16 [14; 16]	0.1
ASA			
I	33 (94.3%)	34 (97.1%)	0.5
II	2 (5.7%)	1 (2.9%)	
Penile Hypospadias Type			0.5
Anterior	23 (32.8%)	27 (38.6%)	
Mean	8 (11.4%)	6 (8.6%)	
Posterior	4 (5.7%)	2 (2.8%)	
Total fentanyl dose (mcg.kg^− 1^)	1 [1 ; 1]	1 [1 ; 1]	1

**Table 2:** Outcomes of the study. Data are expressed as median [25–75 interquartile ranges] or N (%)

	Dexamethasone group (*N* = 35)	Control group (*N* = 35)	P value
Number of patients requiring rescue analgesia	8 (23%)	17 (49%)	0.02
First administration of rescue analgesia (hours)	19 [14; 19]	10 [5; 10]	0.07
Number of patients requiring Nalbuphin boluses for rescue analgesia	0 (0%)	2 (6%)	0.5
Postoperative vomiting (N)	3 (8.5%)	5 (11.4%)	0.2

Postoperatively, patients exhibited urethrocutaneous fistula and cosmetic unfavorable outcome that mandatory surgery (additional file 1). The rate of those complications were not significantly different between the two groups. At one year, cosmetic and functional outcomes were judged adequate by the surgeon.

## Discussion

The main result of the study can be summarized as follows: intravenous dexamethasone (0.15 mg.kg^− 1^) reduced the proportion of patients requiring rescue analgesia and increased the duration and the quality of analgesia provided by pudendal block and delayed the first administration of any rescue analgesia.

Our results differ from those previously published on the pudendal block (without dexamethasone) during hypospadias surgery. Two previous studies found the pudendal block, during hypospadias surgery, to result in only 7 to 20% [7, 8] of patients in whom pudendal block was performed still needing complementary analgesia. By contrast, our study found that close to 50% in the control group who had pudendal block without intravenous dexamethasone. This difference might be related to the absence of any administration of anticipatory analgesics at the end of surgery and/or the use of other systemic analgesics such as non-steroidal anti-inflammatory drugs and/or tramadol [7, 17]. The low volume of local anesthetics used might also account for the difference in rescue analgesia need observed between our study and others. For example, the Kendigelen et al. study used 0.25 mL.kg^− 1^/side of more dilute local anesthetics (0.25% bupivacaine, max 10 mL) [7]. The low volume of local anesthetics used in our study might had provided insufficient analgesia leading to a more need for postoperative analgesia.

In accordance with previously published studies in both adults and children [9–12, 18], the current study found intravenous dexamethasone, to decrease and delay the need for rescue analgesia after regional analgesia. The consistency of results concerning these outcomes indicate the probable benefit of systemic dexamethasone as an adjunct to pudendal block during hypospadias surgery.

Most studies, exploring the dexamethasone analgesic effect, have been published using caudal analgesia [13, 18, 19]. These studies have also demonstrated the efficacy of dexamethasone administration by either intravenous or peripheral route for decreasing rescue analgesia and delaying the time for the first rescue analgesia administration. Concerning the efficacy of dexamethasone as an adjunct to peripheral analgesia in children, evidence is more conflicting. Arafa et al. [10] found dexamethasone 0.1 mg.kg^− 1^, administered either by intravenous or perineural route, to decrease the need to rescue analgesia during quadratus lumborum block in children undergoing renal surgery. By contrast, Veneziano et al. [20] did not found such a result when exploring perineural dexamethasone (0.1 mg.kg^− 1^) during femoral block analgesia in children undergoing knee arthroscopy. Dexamethasone dosage differences may explain some of such varied findings. This is supported by the fact that efficacy of this compound in association with caudal analgesia involved smaller perineural doses (ranging from 0.1 to 0.2 mg.kg^− 1^) when compared with the often higher studied intravenous doses ranging from 0.5 to 1.5 mg.kg^− 1^ [11]. Given the demonstrated efficacy of the low dose intravenous dexamethasone observed in both our study and another published by Arafa et al. [10], one can hypothesize that the location of the regional anesthesia could influence upon the dose intravenous dexamethasone necessary to obtain the same effects as the perineural administration [21, 22].

Our study did not demonstrate any opioid-sparing effect during the postoperative period. Although, this can be related to the low intensity of pain associated with hypospadias surgery [21], one cannot exclude the lack of power of the current study for exploring this specific outcome. Otherwise, the high dosage of fentanyl during the induction of anesthesia might have blunt the opioid-sparing effect of dexamethasone.

In addition to its effects on the need for rescue analgesia and the time for the first administration of any rescue analgesia, dexamethasone improved postoperative pain scores. However, as previously found in adult studies [21], this effect was moderate (median difference = 1 in the current studies versus 0.5 on a 1 to 10 scale in adults) [21] and question its real relevance. Interestingly, this effect was only observed between 3 and 24 h after surgery in our study and was similar to previous results published by Arafa et al. [10]. By contrast, previous studies of orchiopexy patients undergoing caudal analgesia [12, 13] have found both intravenous or perineural dexamethasone to improve the quality of analgesia provided by caudal analgesia during the first 3 postoperative hours. In addition, a meta-analysis found dexamethasone to improve analgesia provided by caudal analgesia during the first 6 postoperative hours [11]. Accordingly, our results suggest that the potentiating effect of dexamethasone on the quality of analgesia provided by the pudendal block seems more prolonged with this block in comparison to caudal block.

Although, most studies have found similar effects between perineural and systemic dexamethasone [9, 11, 23], one study in a pediatric surgical population suggested a greater efficacy for perineural dexamethasone when comparison to intravenous dexamethasone, although not to a level of statistical significance [10]. The question is raised, therefore, whether perineural dexamethasone could be more effective than its systemic dexamethasone with respect to postoperative pain outcomes. However, given the antiemetic effect of dexamethasone, in addition to its analgesic effect, intravenous administration of dexamethasone seems to provide the broader effects that largely counterbalance any potential small reduction in quality of analgesia [23].

Dexamethasone was not associated with a reduction in the incidence of vomiting in the current study. A lack of power might account for this negative result. However, the high dosage of fentanyl during the induction of anesthesia might account for this result. This is supported by the high incidence of vomiting in our study in both groups in comparison to published literature. Naja et al. found an incidence of postoperative nausea and vomiting of 1% when using smaller dosage of fentanyl during induction of anesthesia (1 mcg.kg^− 1^).

Although, there is some concern about the effects of corticosteroids on the healing of surgical wound, evidences from literature have not found such effects. A 2019 Cochrane systematic.

review and meta-analysis of 37 trials found no evidence that dexamethasone increased the risk of a postoperative wound healing (OR, 0.99 [95% CI, 0.28 to 3.43]) [24].

The current study suffers some limitations. Firstly, the sample size was insufficient to explore secondary outcomes such as opioid-sparing. However, given the infrequent need for opioid rescue following pudendal block for hypospadias surgery [7, 8], and the moderate opioid-sparing effect of dexamethasone [21] it appears unlikely that exploring this outcome will lead to relevant results. Secondly, the study duration was limited to the initial postoperative 24 h. Second, the current study explored all forms of hypospadias with various surgical technique. Although all studies investigating analgesia technique did not take account of this potential heterogeneity, the efficacy of dexamethasone might be different according to surgery performed. Finally, this study was monocentric and should be replicated across other centers before firm conclusions can be drawn.

In conclusion, a single intravenous dose (0.15 mg.kg^− 1^) of dexamethasone reduces the proportion of patients requiring rescue analgesia and delays the administration of the first dose of rescue analgesia during pudendal block for hypospadias surgery. In addition, a delayed (> 3 h) improvement in pain scores were associated with this effect. Further studies to confirm this effect are needed given the limited number of studies and varied results regarding intravenous dexamethasone in association with regional analgesia in children.

### Electronic supplementary material

Below is the link to the electronic supplementary material.

Supplementary Material 1

## Data Availability

The datasets generated and/or analysed during the current study are not publicly available due to absence of authorization from the hospital management but are available from the corresponding author on reasonable request.

## Footnotes

1 https://clincalc.com/stats/samplesize.aspx.
