# Supplementary material for: The potentiating effect of intravenous dexamethasone upon preemptive pudendal block analgesia for hypospadias surgery in children managed with Snodgrass technique: a randomized controlled study: Dexamethasone for pain management in children
Source: BMC Anesthesiol. 2024 Apr 16;24:145. doi: 10.1186/s12871-024-02536-3 (PMC11020812; doi:10.1186/s12871-024-02536-3)
Supplement: Supplementary file 1 — Supplementary Material 1 [file 12871_2024_2536_MOESM1_ESM.docx]

Supplemental File 1 : Postoperative complications

|  | Dexamethasone group  (N = 35) | Control group  (N = 35) | P value |
| --- | --- | --- | --- |
| Urethrocutaneous fistula | 5 (14.3 %) | 4 (11.4 %) | 1 |
| Cosmetic complications | 1 (2.9 %) | 0 (0 %) | 1 |
| Overall complications | 6 (17.1 %) | 4 (11.4 %) | 0.7 |
